# Supplementary material for: Skeletal muscle PGC-1α1 reroutes kynurenine metabolism to increase energy efficiency and fatigue-resistance
Source: Nat Commun. 2019 Jun 24;10:2767. doi: 10.1038/s41467-019-10712-0 (PMC6591322; doi:10.1038/s41467-019-10712-0)
Supplement: Supplementary file 1 — Supplementary Information [file 41467_2019_10712_MOESM1_ESM.docx]

Supplementary Information for

Skeletal Muscle PGC-1α1 Reroutes Kynurenine Metabolism to Increase Energy Efficiency and Fatigue-Resistance

**Authors:** Leandro Z. Agudelo^1,5^ †, Duarte M.S. Ferreira^1,^†, Shamim Dadvar^1^†, Igor Cervenka^1^, Lars Ketscher^1^, Manizheh Izadi^1^, Liu Zhengye^2^, Regula Furrer^3^, Christoph Handschin^3^, Tomas Venckunas^4^, Marius Brazaitis^4^, Sigitas Kamandulis^4^, Johanna T. Lanner^2^, and Jorge L. Ruas^1,^*

correspondence to: [jorge.ruas@ki.se](mailto:jorge.ruas@ki.se)

Supplementary Figures


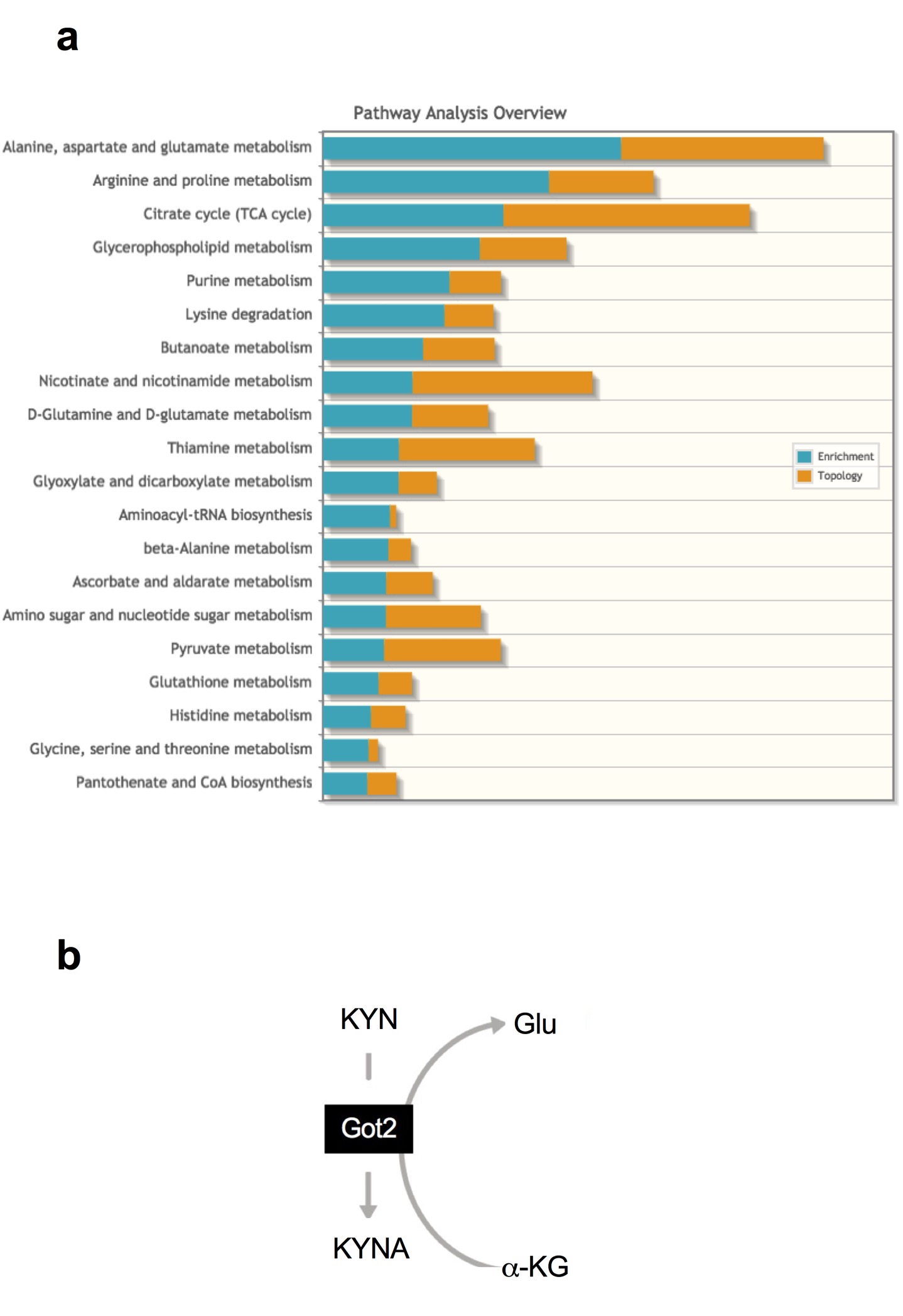


Supplementary Fig. 1. Overview of metabolic pathways under PGC-1α1 control in skeletal muscle. (a) Analysis of metabolic pathways affected by PGC-1α1 expression in skeletal muscle. (b) Schematic representation of kynurenine (Kyn) conversion to Kynurenic acid (Kyna) by GOT2/KAT4.

**
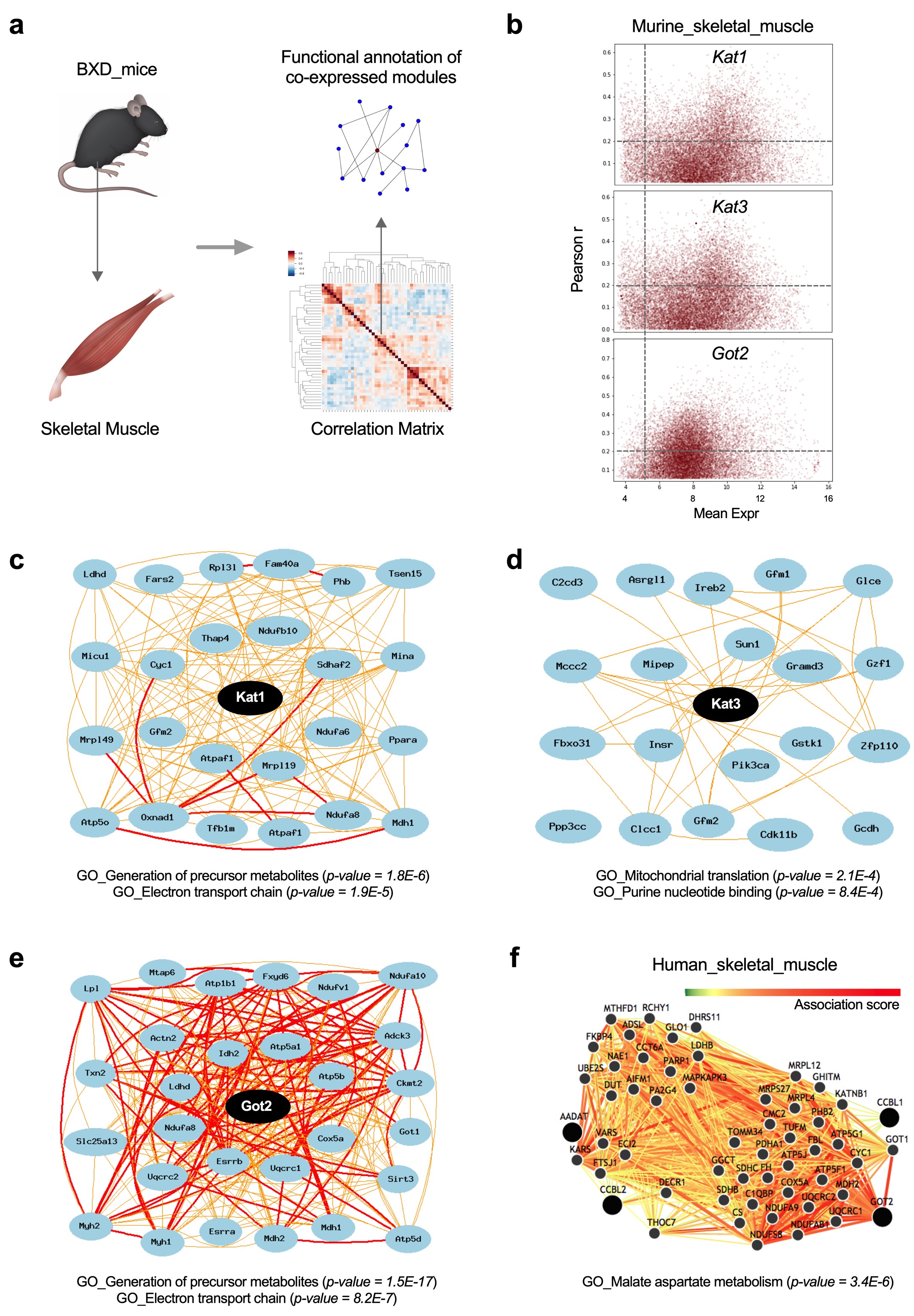
**

**Supplementary Fig. 2. Kat-associated gene networks in murine and human skeletal muscle**. **(a)** Schematic representation for skeletal muscle gene modules co-expressed in BXD mouse strains. **(b)** Pearson correlation of *Kat* enzymes with highly expressed genes in skeletal muscle. We used RNA sequencing data from the Genotype-Tissue Expression project (archived at http://www.genenetwork.org/). **(c)** Gene ontology of *Kat1* co-expressed network. **(d)** Gene ontology of *Kat3* co-expressed network (**e**) Gene ontology of *Got2/Kat4* co-expressed network. **(f)** Tissue-specific gene interactions for the *KAT* enzymes in human skeletal muscle (archived in <http://giant.princeton.edu>)


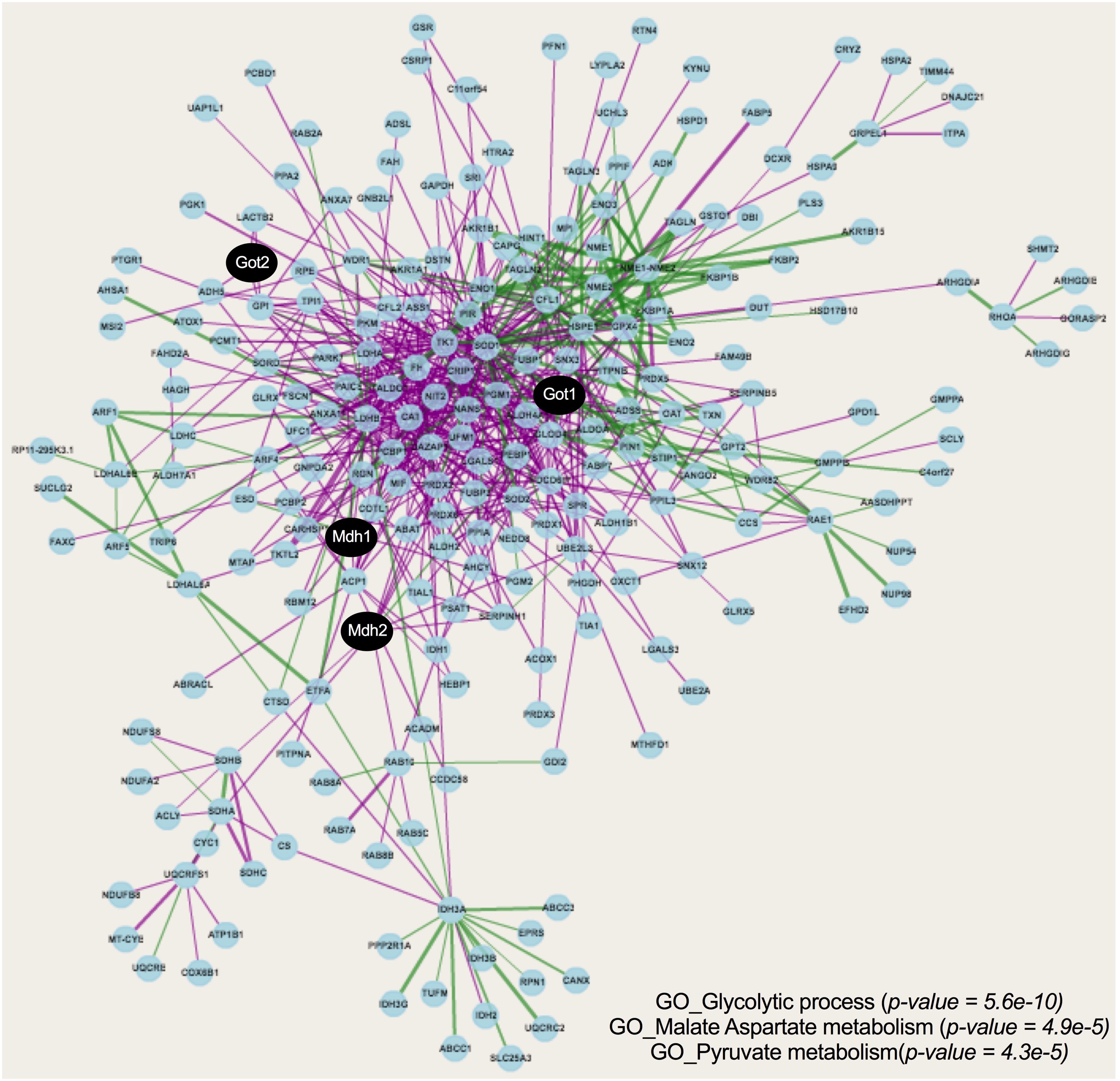


Supplementary Fig. 3. Multiprotein networks associated with the malate-aspartate shuttle. Gene ontology of the malate-aspartate interacting protein-network across metazoan (data stored in <http://metazoa.med.utoronto.ca>)

**
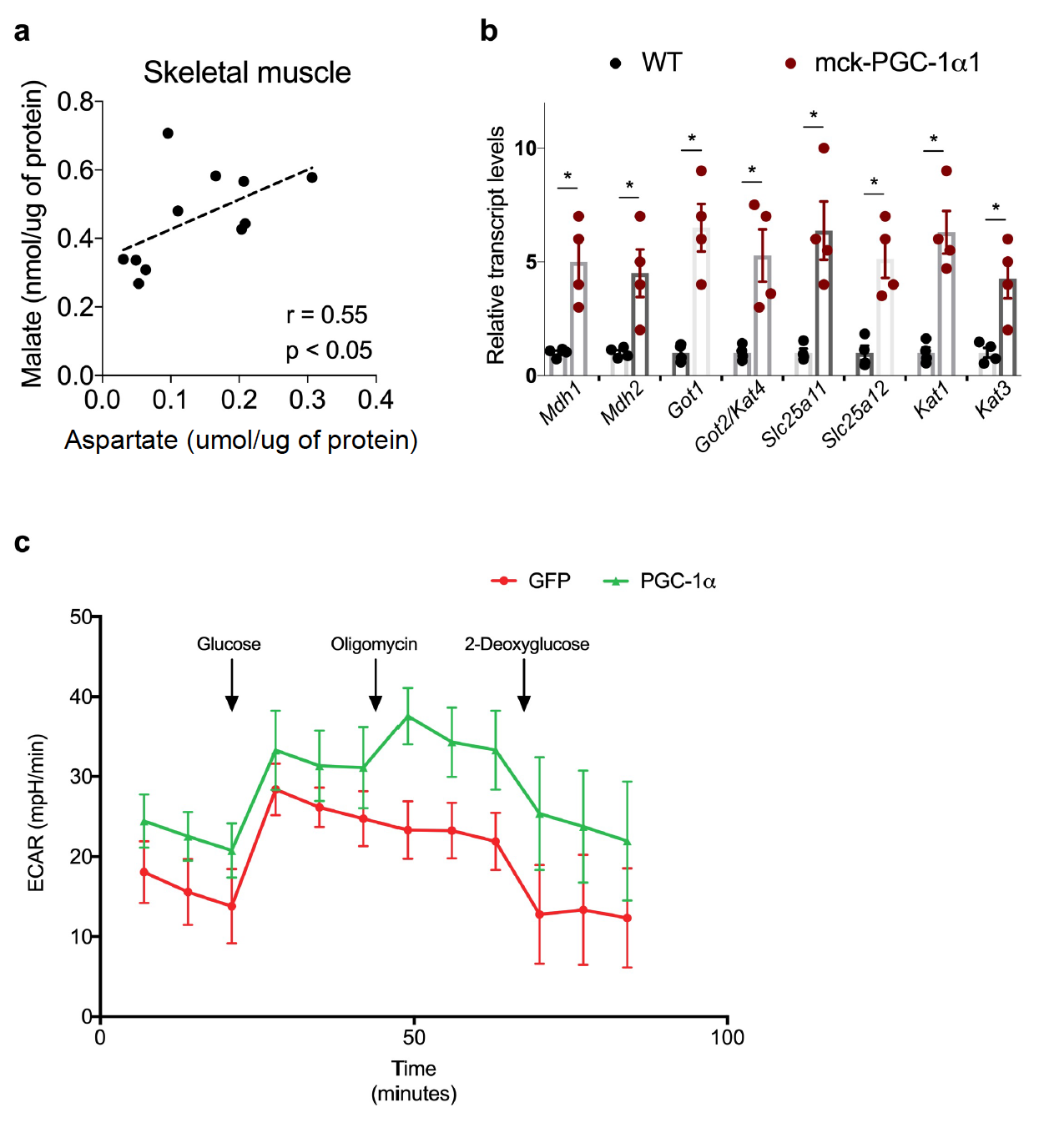
**

**Supplementary Fig. 4.** **Skeletal muscle malate-aspartate correlation**. **(a)** Pearson correlation of aspartate and malate levels in murine skeletal muscle. **(b)** Relative transcript levels of genes involved in malate-aspartate metabolism in skeletal muscle of wild-type (wt) and mck-PGC-1α1 mice (n = 4). **(c)** Extracellular acidification rate (ECAR) of the glycolytic stress test in primary myotubes transduced with *Gfp* control or *Pgc-1α1* adenovirus (n = 4). Bars depict mean values and error bars indicate SEM. Unpaired, two-tailed student’s t-test was used when two groups were compared, and one-way analysis of variance (ANOVA) followed by Fisher’s least significance difference (LSD) test for *post hoc* comparisons were used to compare multiple groups, * p < 0.05.

**
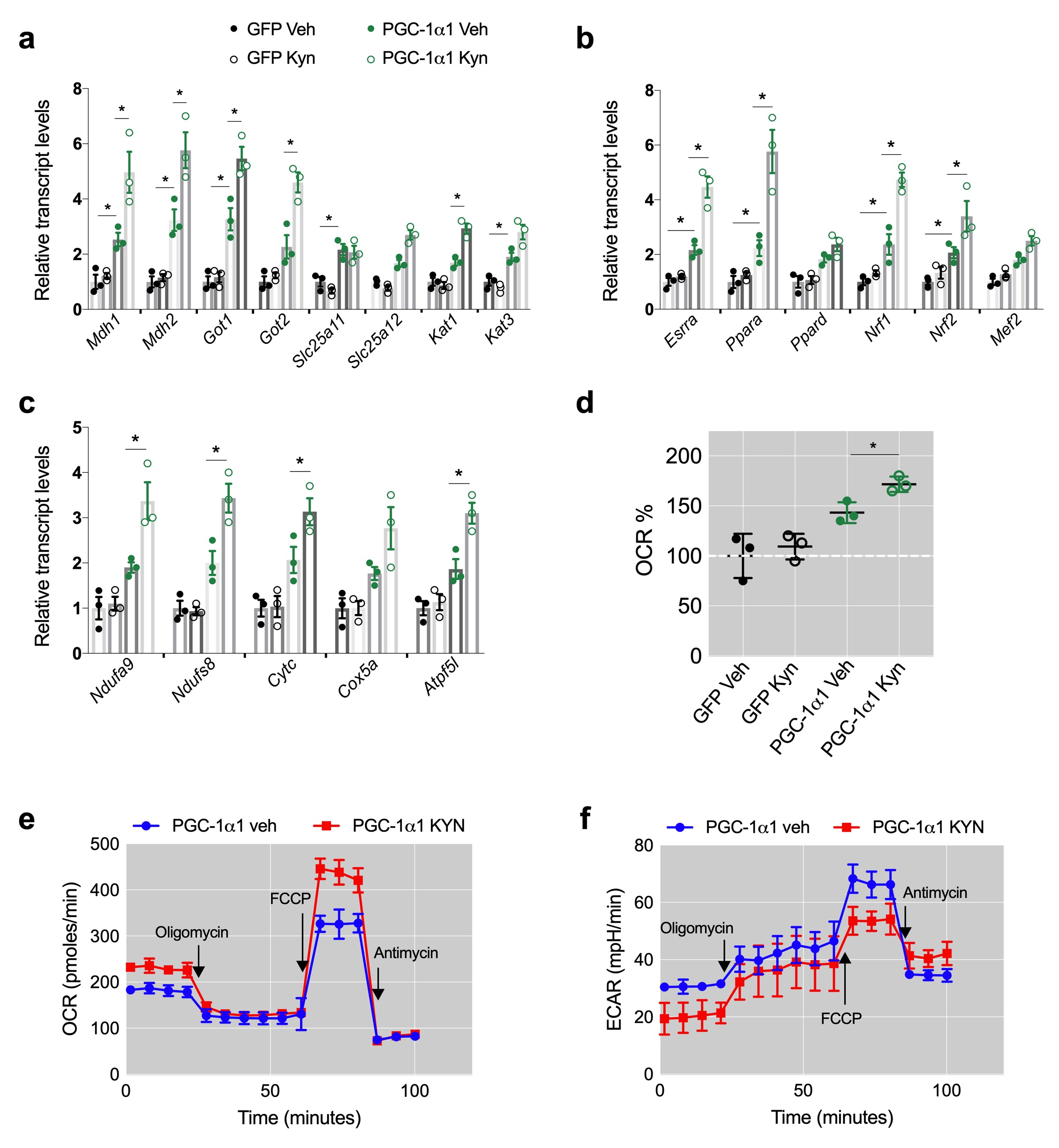
**

**Supplementary Fig. 5. PGC-1α1 myotubes use Kyn to support the malate-aspartate shuttle and mitochondrial respiration.** **(a)** Expression of malate-aspartate shuttle genes in primary myotubes transduced with *Gfp* control or *Pgc-1α1* adenovirus and supplemented with 1 μM kynurenine (Kyn) (n = 3). **(b)** Expression of transcription factors associated with PGC-1α1 function in the same conditions as in (a). **(c)** Expression of mitochondrial genes in the same conditions as in (a). **(d)** Percentage of basal oxygen consumption rate in primary myotubes in the same conditions as in (a) (n = 4). **(e)** Oxygen consumption rates (OCR) in primary myotubes transduced with *Pgc-1α1* adenovirus and supplemented with 1 μM Kyn or PBS control (veh) (n = 4). **(f)** Extracellular acidification rate (ECAR) in the same conditions as in (e). Bars depict mean values and error bars indicate SEM. Unpaired, two-tailed student’s t-test was used when two groups were compared, and one-way analysis of variance (ANOVA) followed by Fisher’s least significance difference (LSD) test for *post hoc* comparisons were used to compare multiple groups, * p < 0.05.


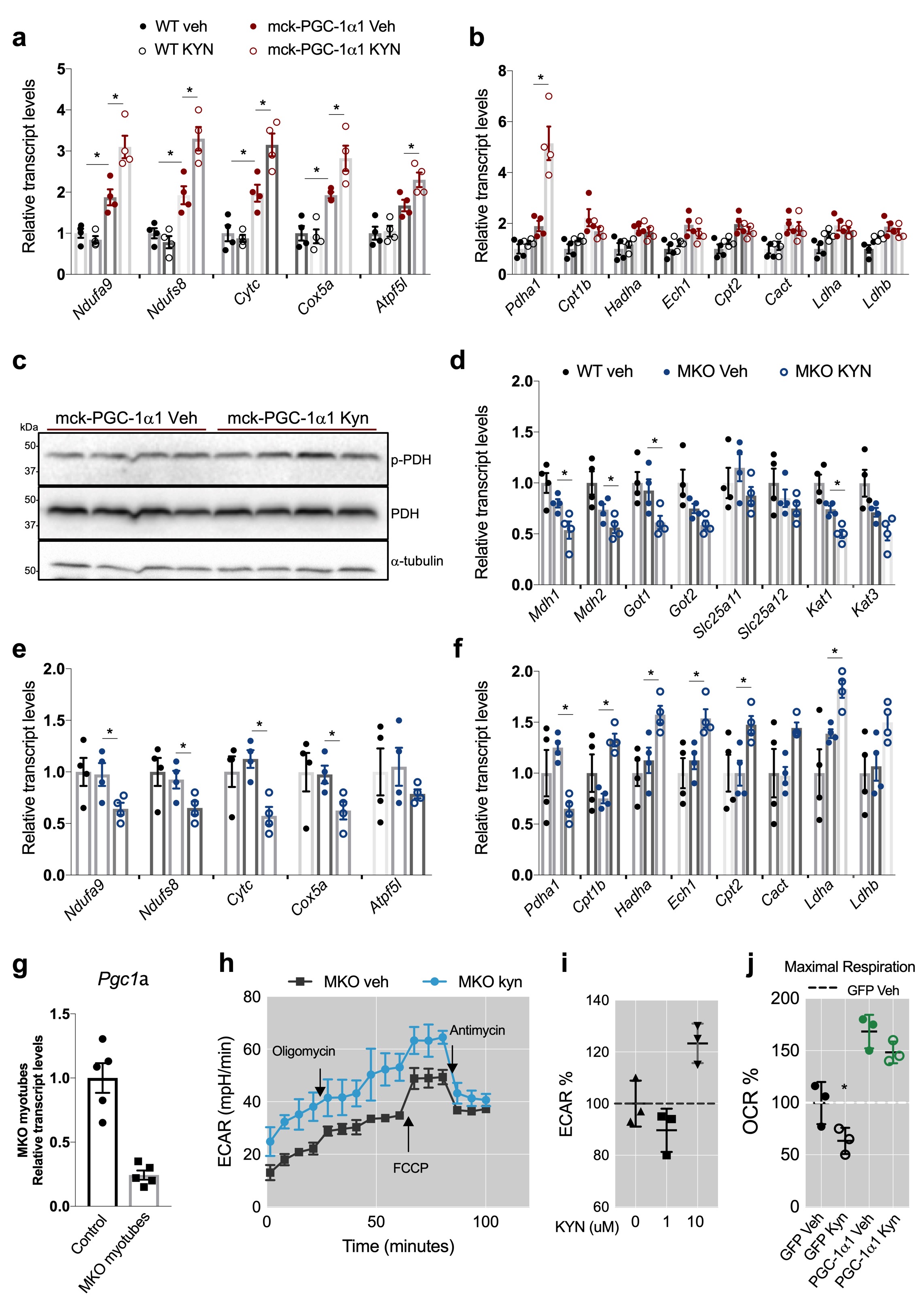


Supplementary Fig. 6. Skeletal muscle PGC-1α1 reroutes Kyn to support the malate-aspartate shuttle. (a) Expression of mitochondrial genes in skeletal muscle of wild-type (wt) and mck-PGC-1α1 mice with a single dose of kynurenine (Kyn, 2.5 mg/kg) (n = 4). (b) Expression of genes integrating glucose oxidation (*Pdha1*) and fatty acid oxidation in the same conditions as (a). (c) Representative blot of PDH and p-PDH in skeletal muscle of mck-PGC-1α1 mice in the same conditions as (a). (d) Relative transcript expression of genes involved in malate-aspartate metabolism in skeletal muscle of MKO-PGC-1α1 mice with a single intraperitoneal dose of Kyn (2.5 mg/kg) (n = 4). (e) Expression of mitochondrial genes in skeletal muscle of wt and MKO-PGC-1α1 mice in the same conditions as (d). (f) Expression of genes integrating glucose oxidation (*Pdha1*) and fatty acid oxidation in the same conditions as (d). (g) Relative transcript levels of *Pgc-1α1* in primary myotubes from wt or MKO-PGC-1α1 mice (n = 5). (h) Extracellular acidification rate (ECAR) in primary myotubes from wt or MKO-PGC-1α1 mice supplemented with 1 μM Kyn for 4 h. (i) Percentage of ECAR in primary myotubes from MKO-PGC-1α1 mice supplemented with 0, 1 or 10 μM Kyn (n = 3). (j) Percentage of oxygen consumption rates (OCR) at maximal respiration in primary myotubes transduced with *Gfp* control or *Pgc-1α1* adenovirus supplemented with 10 μM Kyn (n = 3). Bars depict mean values and error bars indicate SEM. Unpaired, two-tailed student’s t-test was used when two groups were compared, and one-way analysis of variance (ANOVA) followed by Fisher’s least significance difference (LSD) test for *post hoc* comparisons were used to compare multiple groups, * p < 0.05.


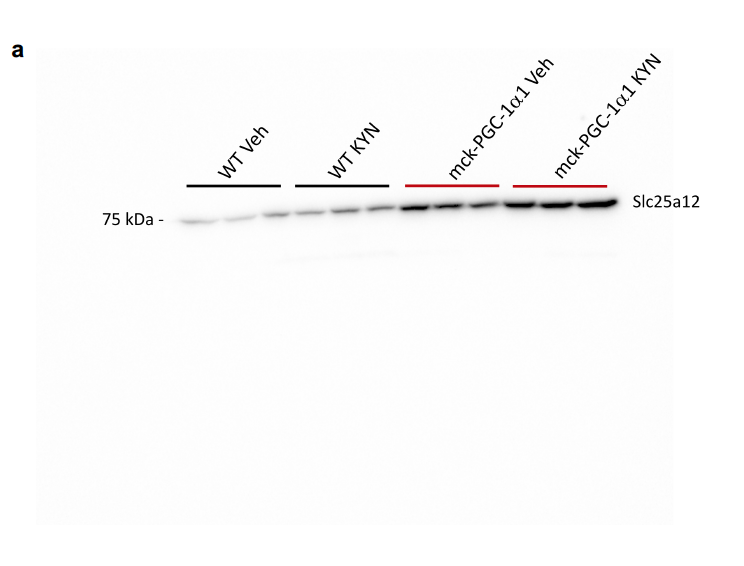
**
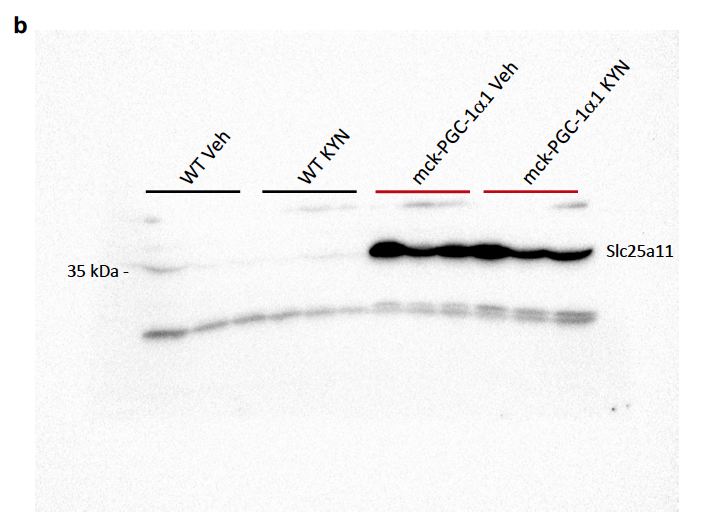
**


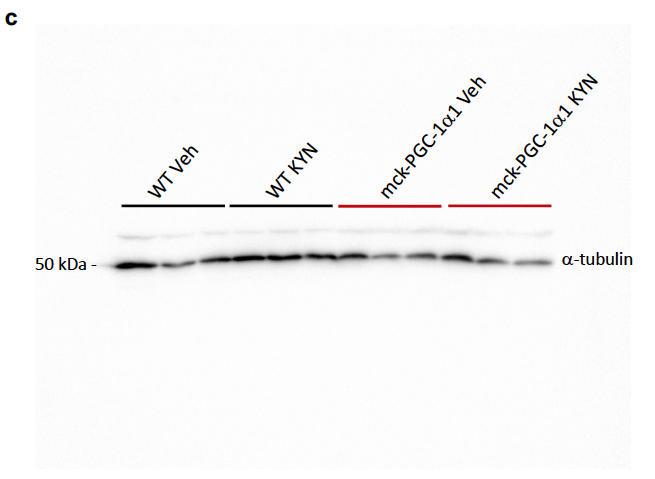


**Supplementary Fig. 7**. **Uncropped western blots shown in Fig. 2c.**

Protein levels of the malate-aspartate shuttle constituents SLC25A11 and SLC25A12 in skeletal muscle of wt and mck-PGC-1α1 mice with a single intraperitoneal dose of Kyn (2.5 mg/kg).


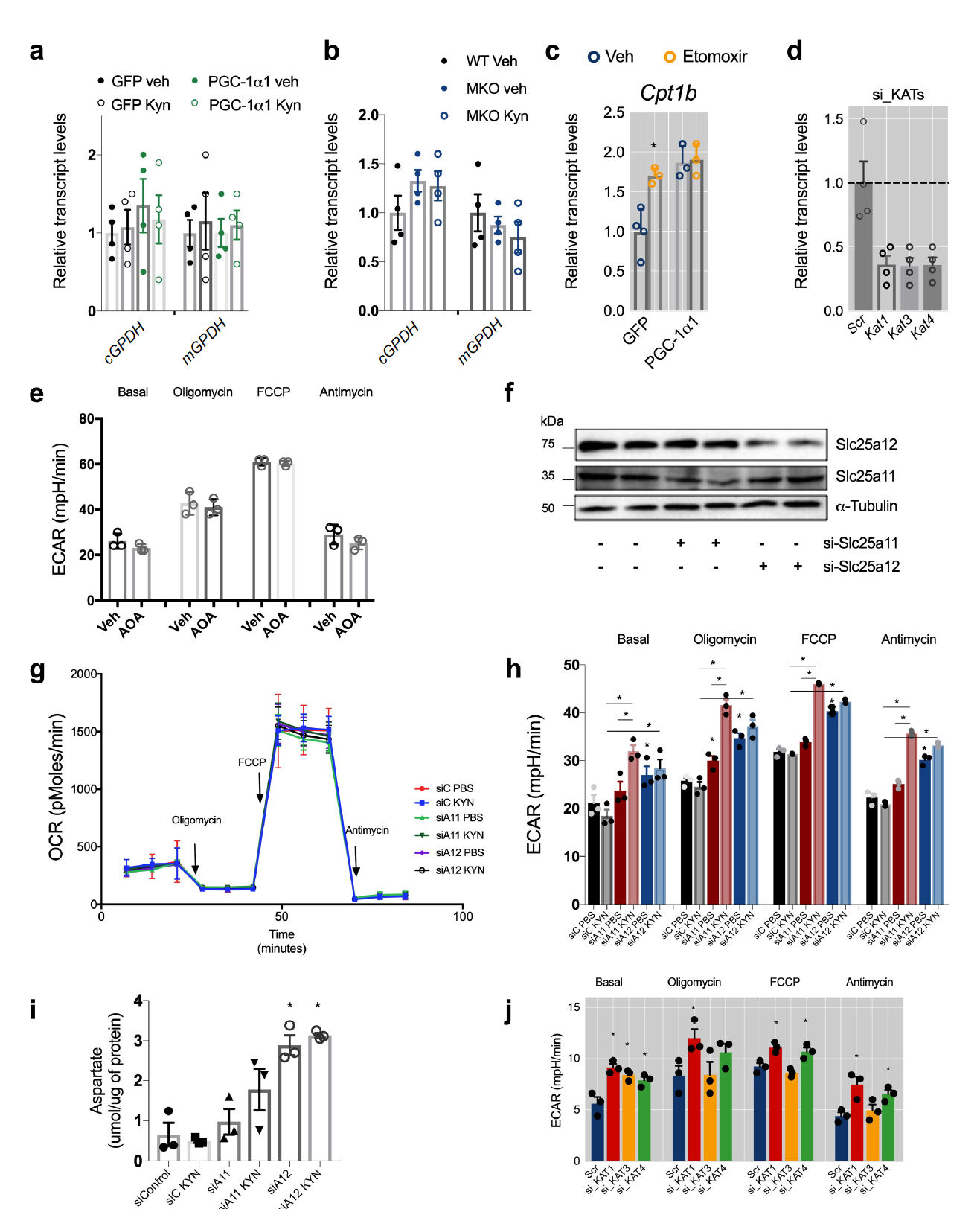


Supplementary Fig. 8. Effects on myotubes upon Etomoxir treatment or *Slc25a11*, *Slc25a12* or *Kat* gene expression silencing. (a) Relative transcript levels of glycerol-3-phosphate shuttle genes in primary myotubes transduced with *Gfp* control or *Pgc-1α1* adenovirus and supplemented with 1 μM Kyn (n = 4). (b) Relative transcript levels of glycerol-3-phosphate shuttle genes in skeletal muscle of MKO-PGC-1α1 mice with a single intraperitoneal dose of Kyn (25 mg/Kg) (n = 4). (c) *Cpt1b* mRNA expression in primary myotubes transduced with *Gfp* control or *Pgc-1α1* adenovirus and supplemented with 50 μM Etomoxir for 1 h (n = 3-4). (d) *Kat1*, *Kat3* and *Got2/Kat4* mRNA expression in primary myotubes transfected with scrambled siRNA or *Kat*-specific siRNA (n = 4). (e) Extracellular acidification rate (ECAR) in primary myotubes supplemented with 100 μM Aminooxyacetate (AOA) for 1 h (n = 3). (f) Representative blot of SLC25A11 and SLC25A12 in differentiated primary myotubes transfected with scrambled siRNA or *Slc25a11*- or *Slc25a12*-specific siRNA. (g) Oxygen consumption rates (OCR) after Mitochondrial Stress Test in differentiated primary myotubes in the same conditions as (f) and with or without 1 μM Kyn supplementation for 4 h (n = 3). (h) ECAR after Mitochondrial Stress Test in differentiated primary myotubes in the same conditions as (g). (i) Aspartate levels normalized by protein content in differentiated primary myotubes in the same conditions as (g). (j) ECAR after Mitochondrial Stress Test in differentiated primary myotubes in the same conditions as (d). Bars depict mean values and error bars indicate SEM. Unpaired, two-tailed student’s t-test was used when two groups were compared, and one-way analysis of variance (ANOVA) followed by Fisher’s least significance difference (LSD) test for *post hoc* comparisons were used to compare multiple groups, * p < 0.05.


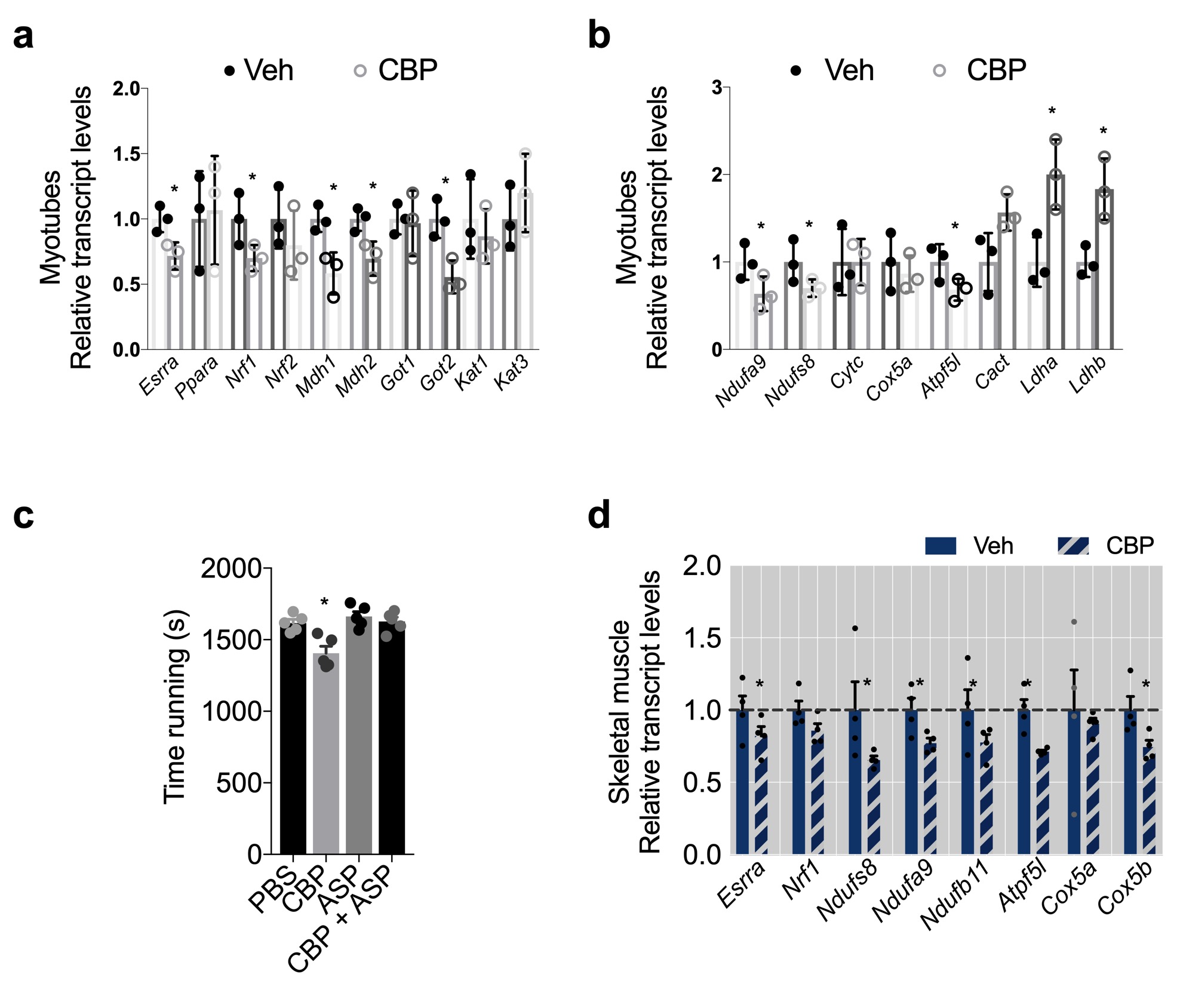


Supplementary Fig. 9. Carbidopa (CBP) treatment impairs muscle energy metabolism. (a) Expression of energy metabolism genes in primary myotubes supplemented with 40 μM CBP for 24 h (n = 3). (b) Expression of mitochondrial genes in the same conditions as in (a). (c) Exercise performance test expressed as maximal run time after 4 days of intraperitoneal injections of PBS, CBP, aspartate (ASP) or CBP + ASP (n = 5). (d) Expression of mitochondrial genes in mouse skeletal muscle after 4 days of intraperitoneal injections of PBS (Veh) or CBP (n = 4). Bars depict mean values and error bars indicate SEM. Unpaired, two-tailed student’s t-test was used when two groups were compared, and one-way analysis of variance (ANOVA) followed by Fisher’s least significance difference (LSD) test for *post hoc* comparisons were used to compare multiple groups, * p < 0.05.


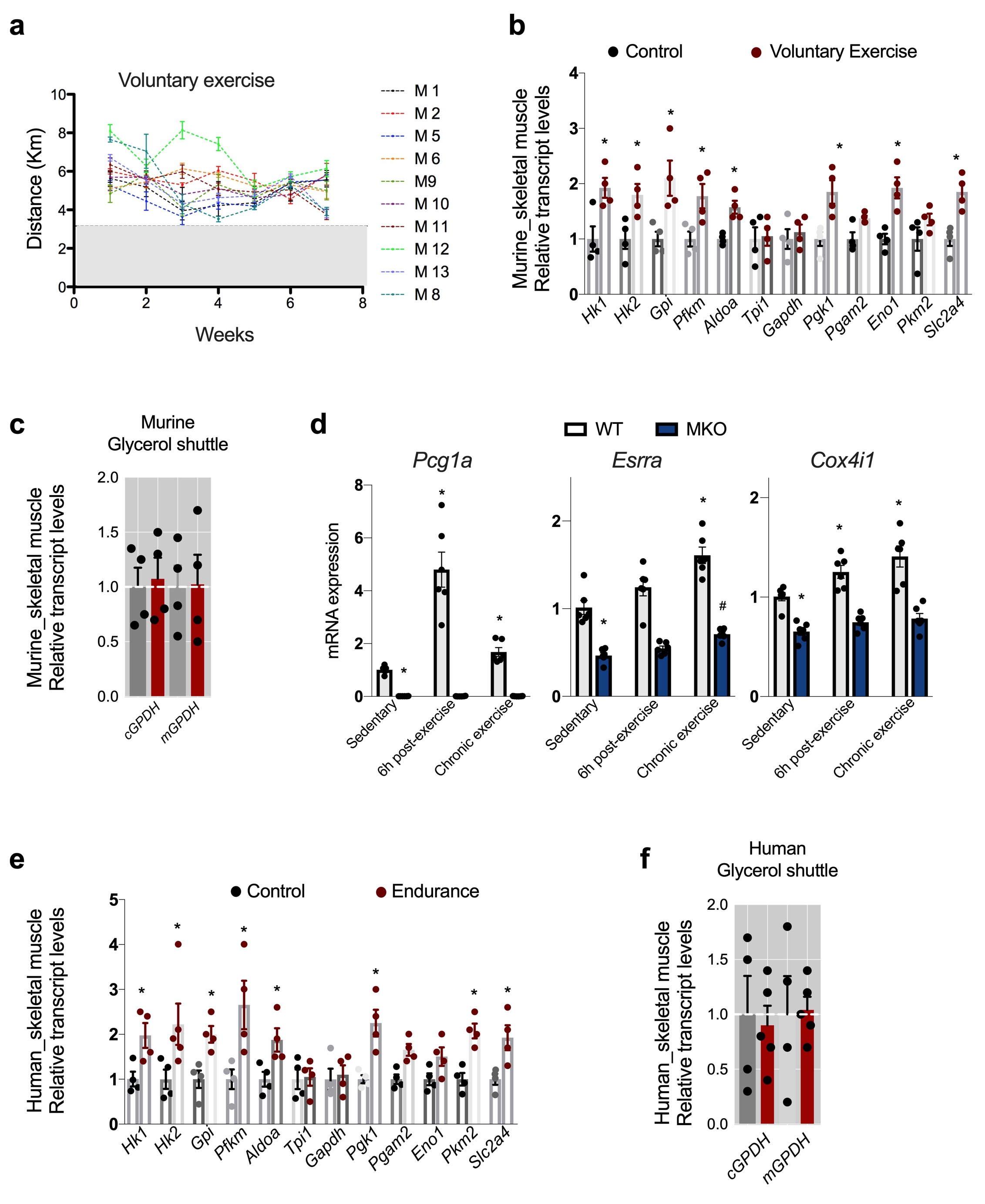


Supplementary Fig. 10. Endurance exercise increases transcript levels of genes involved in glycolysis in both murine and human skeletal muscle. (a) Running distance (km) of wild-type (wt) mice with free access to running wheels for 7 weeks. (b) Relative transcript levels of genes involved in glycolysis in skeletal muscle of sedentary wild-type mice and wild-type mice with access to free-wheel running for 8 weeks (n = 4). (c) Relative levels of glycerol shuttle in the same conditions as (b). (d) Relative transcript levels of *Pgc-1α1, Esrra*, and *Cox4i1* in skeletal muscle of sedentary, acutely and chronically exercised wt and MKO-PGC-1α1 mice. (n = 6) (e) Expression of genes involved in glycolysis in skeletal muscle of human volunteers after endurance exercise (n = 4-5). (f) Relative levels of glycerol shuttle in the same conditions as (d). Bars depict mean values and error bars indicate SEM. Unpaired, two-tailed student’s t-test was used, * p < 0.05.

Supplementary Table 1. Primer sequences used for RT-qPCR.

Supplementary Table 2. mck-PGC-1α1 transcriptomics

Skeletal-muscle transcriptomic profile from mck-PGC-1α1 transgenic mice.

Use the "Insert Citation" button to add citations to this document.
