# Supplementary material for: Skeletal muscle PGC-1α1 reroutes kynurenine metabolism to increase energy efficiency and fatigue-resistance
Source: Nat Commun. 2019 Jun 24;10:2767. doi: 10.1038/s41467-019-10712-0 (PMC6591322; doi:10.1038/s41467-019-10712-0)
Supplement: Supplementary file 6 — Reporting Summary [file 41467_2019_10712_MOESM6_ESM.pdf]

nature research

Corresponding author(s): Jorge Ruas

Last updated by author(s): Apr 8, 2019

Reporting Summary

Nature Research wishes to improve the reproducibility of the work that we publish. This form provides structure for consistency and transparency in reporting. For further information on Nature Research policies, see Authors & Referees and the Editorial Policy Checklist.

Statistics

For all statistical analyses, confirm that the following items are present in the figure legend, table legend, main text, or Methods section.

n/a	Confirmed	
☐	☒	The exact sample size (n) for each experimental group/condition, given as a discrete number and unit of measurement
☐	☒	A statement on whether measurements were taken from distinct samples or whether the same sample was measured repeatedly
☐	☒	The statistical test(s) used AND whether they are one- or two-sided<br>*Only common tests should be described solely by name; describe more complex techniques in the Methods section.*
☒	☐	A description of all covariates tested
☐	☐	A description of any assumptions or corrections, such as tests of normality and adjustment for multiple comparisons
☐	☒	A full description of the statistical parameters including central tendency (e.g. means) or other basic estimates (e.g. regression coefficient) AND variation (e.g. standard deviation) or associated estimates of uncertainty (e.g. confidence intervals)
☐	☒	For null hypothesis testing, the test statistic (e.g. F, t, r) with confidence intervals, effect sizes, degrees of freedom and P value noted<br>*Give P values as exact values whenever suitable.*
☒	☐	For Bayesian analysis, information on the choice of priors and Markov chain Monte Carlo settings
☒	☐	For hierarchical and complex designs, identification of the appropriate level for tests and full reporting of outcomes
☐	☒	Estimates of effect sizes (e.g. Cohen's d, Pearson's r), indicating how they were calculated

Our web collection on statistics for biologists contains articles on many of the points above.

Software and code

Policy information about availability of computer code

Data collection	Please see the information provided in the methods section of the manuscript.
Data analysis	Please see the information provided in the methods section of the manuscript.

For manuscripts utilizing custom algorithms or software that are central to the research but not yet described in published literature, software must be made available to editors/reviewers. We strongly encourage code deposition in a community repository (e.g. GitHub). See the Nature Research guidelines for submitting code & software for further information.

Data

Policy information about availability of data

All manuscripts must include a data availability statement. This statement should provide the following information, where applicable:

- Accession codes, unique identifiers, or web links for publicly available datasets
- A list of figures that have associated raw data
- A description of any restrictions on data availability

Accession codes and web-links for publicly available datasets are provided in the manuscript.

Field-specific reporting

Please select the one below that is the best fit for your research. If you are not sure, read the appropriate sections before making your selection.

☒ Life sciences ☐ Behavioural & social sciences ☐ Ecological, evolutionary & environmental sciences

For a reference copy of the document with all sections, see nature.com/documents/nr-reporting-summary-flat.pdf

October 2018

Life sciences study design

All studies must disclose on these points even when the disclosure is negative.

Sample size	Sample sizes were used based on previous experience and standards within the field to have sufficient number of samples to be able to reach statistical significance.
Data exclusions	No data was excluded.
Replication	Number of replicates were used based on previous experience and standards within the field to have sufficient number of samples to be able to reach statistical significance.
Randomization	All mice used were of the same age, gender and litters. For experiments, mice were weight-matched.
Blinding	Investigators were blinded to genotype but not to treatment due to technical limitations.

Reporting for specific materials, systems and methods

We require information from authors about some types of materials, experimental systems and methods used in many studies. Here, indicate whether each material, system or method listed is relevant to your study. If you are not sure if a list item applies to your research, read the appropriate section before selecting a response.

Materials & experimental systems

n/a	Involved in the study	
☐	☒	Antibodies
☒	☐	Eukaryotic cell lines
☒	☐	Palaeontology
☐	☒	Animals and other organisms
☐	☒	Human research participants
☒	☐	Clinical data

Methods

n/a	Involved in the study	
☒	☐	ChIP-seq
☒	☐	Flow cytometry
☒	☐	MRI-based neuroimaging

Antibodies

Antibodies used	Monoclonal antibodies anti-PDH-E1a(9H9AF5; Thermo Fisher Scientific), anti-Slc25a12 (kind gift of Dr. Jorgina Satrústegui)<br><br>Polyclonal antibodies anti-phosphoPDH-E1a Ser290 (AP1062; Calbiochem), anti-Slc25a11 (AB80464; Abcam).<br><br>Monoclonal antibody anti-a-tubulin (T6199; Sigma-Aldrich).
Validation	Validation of the above mentioned antibodies are available on the manufacturer's website.

Animals and other organisms

Policy information about studies involving animals; ARRIVE guidelines recommended for reporting animal research

Laboratory animals	Mus musculus. All males, ages 10-14 weeks. C57BL/6J mice were used as wt controls. Mck-PGC-1α1 and MKO-PGC-1α1 mice were all on a C57bl/6J background.
Wild animals	The study did not involve wild animals.
Field-collected samples	The study did not involve field-collected samples.
Ethics oversight	All experiments were approved by the regional animal ethics Committee of Northern Stockholm, Sweden or of Basel-Stadt, Switzerland.

Note that full information on the approval of the study protocol must also be provided in the manuscript.

Human research participants

Policy information about studies involving human research participants

Population characteristics	All subjects were male. The first group consisted of nine recreationally active subjects who were pursuing endurance training for a 150-km road cycling time trial. The subjects of the second group were also recreationally active but did not specifically participate in endurance training.

Recruitment	Subjects were recruited locally through advertisement.
Ethics oversight	The study was approved by the local ethics committee of Kaunas, Lithuania.

Note that full information on the approval of the study protocol must also be provided in the manuscript.
