# Supplementary material for: Skeletal muscle PGC-1α1 reroutes kynurenine metabolism to increase energy efficiency and fatigue-resistance
Source: Nat Commun. 2019 Jun 24;10:2767. doi: 10.1038/s41467-019-10712-0 (PMC6591322; doi:10.1038/s41467-019-10712-0)
Supplement: Supplementary file 7 — Description of Additional Supplementary Files [file 41467_2019_10712_MOESM7_ESM.docx]

**Title: Supplementary Data 1.**
**Description:** mck-PGC-1α1 transcriptomics Skeletal-muscle transcriptomic profile from mck-PGC-1α1 transgenic mice.

**Title: Supplementary Data 2.
Description:**  Primer sequences Primer sequences used for RT-qPCR
